# Natural killer cell NKG2D and granzyme B are critical for allergic pulmonary inflammation^⋆^

**DOI:** 10.1016/j.jaci.2013.09.048

**Published:** 2014-03

**Authors:** Nazanin Farhadi, Laura Lambert, Chiara Triulzi, Peter J.M. Openshaw, Nadia Guerra, Fiona J. Culley

**Affiliations:** a Centre for Respiratory Infection and MRC-Asthma UK Centre for Allergic Mechanisms in Asthma, Department of Respiratory Infections, National Heart and Lung Institute, Faculty of Medicine, Imperial College London, London, United Kingdom; b Division of Cell and Molecular Biology, Faculty of Natural Sciences, Imperial College London, London, United Kingdom

**Keywords:** Innate immunity, lung, natural killer cell, house dust mite, allergic inflammation, asthma, APC, Allophycocyanin, BAL, Bronchoalveolar lavage, HDM, House dust mite, KO, Knock-out, NK, Natural killer, RSV, Respiratory syncytial virus, WT, Wild type

## Abstract

**Background:**

The diverse roles of innate immune cells in the pathogenesis of asthma remain to be fully defined. Natural killer (NK) cells are innate lymphocytes that can regulate adaptive immune responses. NK cells are activated in asthma; however, their role in allergic airway inflammation is not fully understood.

**Objective:**

We investigated the importance of NK cells in house dust mite (HDM)-triggered allergic pulmonary inflammation. Specifically, we aimed to determine the role of the major NK-cell activating receptor NKG2D and NK-cell effector functions mediated by granzyme B.

**Methods:**

Allergic airway inflammation was induced in the airways of mice by repeated intranasal HDM extract administration and responses in wild-type and NKG2D-deficient mice were compared. Adoptive transfer studies were used to identify the cells and mechanisms involved.

**Results:**

Mice that lacked NKG2D were resistant to the induction of allergic inflammation and showed little pulmonary eosinophilia, few airway T_H_2 cells, and no rise in serum IgE after multiple HDM-allergen exposures. However, NKG2D was not required for pulmonary inflammation after a single inoculation of allergen. NKG2D-deficient mice showed no alteration in responses to respiratory virus infection. Transfer of wild-type NK cells (but not CD3^+^ cells) into NKG2D-deficient mice restored allergic inflammatory responses only if the NK cells expressed granzyme B.

**Conclusions:**

These studies established a pivotal role for NK-cell NKG2D and granzyme B in the pathogenesis of HDM-induced allergic lung disease, and identified novel therapeutic targets for the prevention and treatment of asthma.

Asthma is a chronic disease of increasing prevalence that affects 300 million people worldwide.^1,2^ Current therapies are of limited efficacy, so there is a pressing need for further understanding of the immune pathways that drive allergic lung disease. There is a growing interest in the role of innate cells in the initiation of allergic airway inflammation. Allergens, including those of the house dust mite (HDM), *Dermatophagoides pteronyssinus*, the most common trigger of allergic asthma worldwide, can trigger pattern recognition receptors on antigen-presenting cells and on structural cells, such as respiratory epithelial cells,^3-5^ which leads to the activation of innate immune cells, including type-2 innate lymphocytes, γδ T cells, NKT cells, and dendritic cells, which promote T_H_2 immune responses.^6-10^

Natural killer (NK) cells comprise approximately 10% of lung lymphocytes in homeostasis in man and mouse.^11^ They are known to kill transformed and infected cells, and they act as a potent source of IFN-γ, thus promoting T_H_1 responses, but they also can produce pro- and anti-inflammatory cytokines, including IL-5, IL-10, and IL-13.^12-14^ In addition, NK cells can regulate adaptive responses by direct killing of activated leukocytes, including dendritic cells, macrophages, and T cells.^15-18^ NK cells express an array of different activating and inhibitory receptors that exhibit nonredundancy *in vivo,* for example, NKp46 is required for protection against influenza virus infection.^19^ Therefore, NK-cell receptors are attractive potential targets for specific therapies, and, thus, there is a need to better define the roles of individual NK-cell receptors in diverse diseases.

NKG2D is an activating receptor expressed on all mature NK cells, NKT cells, and subsets of γδ and αβ T cells.^20,21^ The NKG2D receptor mediates the “stress surveillance” function of NK cells and recognizes ligands from the H60, MULT-1, and the Rae-1 families in mice, and MHC class I chain-related molecules (MICA or MICB) and UL16-binding proteins in man, which are induced in response to DNA damage and on transformed cells.^22,23^ NKG2D has been implicated in tumor clearance, graft rejection, atherosclerosis, autoimmunity, and infection.^22,24-29^ In murine models, activation of skin intraepithelial lymphocytes via NKG2D can promote systemic atopy.^30^ In severe asthma, peripheral blood NK cells express high levels of NKG2D, which correlates with blood eosinophilia.^31^ Furthermore, NKG2D ligands MICA and ULBP-2 are elevated in the serum of children with respiratory symptoms of HDM allergy.^32^

To explore the role of NKG2D expression by NK cells in the induction and control of atopic lung disease, we studied the inflammatory response after challenge with HDM extract. NK cells were recruited to the lungs and airways in this model, and the NKG2D ligand MULT-1 was selectively upregulated in the lung. Allergic inflammation was severely attenuated in mice deficient in NKG2D but was restored in NKG2D-deficient mice by adoptive transfer of wild-type but not granzyme B deficient NK cells. These data provide evidence that NK cells are critical for enhancing lung inflammation in response to HDM allergen, and they do this via both NKG2D and granzyme B production.

## Methods

### Mice

Female BALB/c, C57BL/6, and granzyme B deficient (*gzmb*^−/−^) mice were purchased from Jackson Labs (Charles River Ltd, Margate, United Kingdom) and were used at 6-8 weeks old. NKG2D deficient (*klrk1*^−/−^) mice, which were on the C57BL/6 background, were genotyped as described.^24^ The animals were housed at the Imperial College London animal facility with food and water *ad libitum*. UK Home Office guidelines for animal welfare based on the Animals (Scientific Procedures) Act 1986 were strictly observed. For experiments that involved NKG2D deficient mice, wild-type control animals were age- and sex-matched littermates (*klrk1*^+/+^).

### Allergen challenge and tissue processing

To induce allergic airway inflammation, the mice were challenged by intranasal instillation of 25 μL of a 1 mg/mL solution of whole HDM extract (Greer Laboratories, Inc, Lenoir, NC) in PBS under isoflurane inhalational anesthesia, 3 times a week for 3 weeks.^33,34^ In other experiments, the mice received a single dose of 70 μg of HDM extract. Control mice received PBS. Responses were analyzed 24 hours after the last challenge. For respiratory syncytial virus (RSV) infection, RSV strain A2 (ATCC, Manassas, Va) was grown in Hep-2 cells. The mice received 10^6^ pfu or control Hep-2 cell lysate, intranasally in 50 μL. The mice were weighed daily, and responses were analyzed 4 days after challenge.

The mice were bled under terminal anesthesia by cardiac puncture to collect blood for serum. Bronchoalveolar lavage (BAL) was performed by flushing the lungs 3 times with 1 mL 5 mM EDTA, PBS via a tracheal cannula. Lung vasculature was perfused *in situ* with PBS via the right atrium. Mediastinal lymph nodes were removed, and single cell suspensions were obtained by passing the nodes through a 100-μm mesh. For histologic analysis, one lobe of lung was inflated with PBS and fixed in 10% normal buffered formalin. Specimens were paraffin embedded, transverse sectioned (4 μm) onto glass slides, and stained with hematoxylin and eosin. Images were recorded by using a ×10 objective lens (Zeiss Axioscope.A1; Carl Zeiss Ltd, Welwyn Garden City, United Kingdom). For PCR, lung tissue was snap frozen in liquid nitrogen. For analysis of the lung cellular response, lung tissue was digested with collagenase XI (Sigma Aldrich Company Ltd, Gillingham, United Kingdom), and single-cell suspensions were obtained by using a gentle MACS dissociator (Milltenyi Biotec Ltd, Woking, United Kingdom). After isolation of leukocytes from each tissue and lysis of erythrocytes in ACK buffer (150 mM ammonium chloride, 10 mM potassium bicarbonate, 0.1 mM EDTA), total cell counts were obtained on a FACSCanto flow cytometer (BD Biosciences, Becton Dickinson UK Limited, Oxford, United Kingdom) by using CountBright counting beads (Life Technologies Ltd, Paisley, United Kingdom). For differential cell counts, BAL leukocytes were applied to glass slides by centrifugation (Shandon Cytospin II; Thermo Fisher Scientific, Loughborough, United Kingdom), fixed, and stained with Quick-Diff (Reagena; International Oy Ltd, Toivala, Finland).

### Flow cytometry

The cells were stained with combinations of the following antibodies. Alexafluor 700 or allophycocyanin (APC)-H7 conjugated mAb to CD4 (GK1.5), Pacific Blue conjugated mAb to CD8 (35-6.7), Alexafluor 700 or PerCP-Cy5.5 conjugated mAb to NKp46 (29A1.4), PE-Cy7 conjugated mAb to IFN-γ (XMG1.2) and FITC conjugated mAb to γδ-TCR (GL3) were purchased from BD Biosciences. APC-efluor780 conjugated mAb to CD3 (17A2), PE conjugated mAb to IL-13 (eBio13A), PE conjugated mAb to NKG2D (CX5), PE-Cy7 mAb to CD69 (H1.2F3), APC-conjugated mAb to IL-4 (11B11), and APC conjugated mAb to NK1.1 (PK136) were purchased from eBioscience (Hatfield, United Kingdom). APC conjugated mAb to Granzyme B (GB12) was purchased from Life Technologies.

For intracellular cytokine staining, cells were first stimulated with 100 ng/mL PMA, 1 μg/mL ionomycin (Sigma Aldrich) in the presence of GolgiStop (BD Biosciences) for 4 hours. The cells were stained with aqua live/dead stain (Life Technologies), followed by staining for surface antigens, fixation, then permeabilization for intracellular staining. Flow cytometric acquisition was performed on a FACS Fortessa (BD Biosciences) by using Diva software and a minimum of 50,000 live, single-cell events were collected. Analysis was carried out by using FlowJo software (Tree Star Inc, Ashland, Ore). Dead cells were gated out, and doublets were excluded by means of forward scatter-width (FSC-W) versus FSC-area (FSC-A).

### Sorting and adoptive transfer

Splenic NK cells (CD3^−^NKp46^+^ lymphocytes) or CD3^+^ lymphocytes from wild type, *klrk1*^−/−^ and *gzmb*^−/−^ mice were sorted on a FACSAria III (Becton Dickenson, UK Ltd, Oxford, United Kingdom). Dead cells and doublets were excluded, and purity of sorted cells was typically 98%. A total of 0.5 × 10^6^ cells were transferred intravenously per mouse 24 hours before the first HDM challenge.

### PCR analysis

Lung tissue was homogenized by using a TissueLyser (Qiagen, Manchester, United Kingdom), and total RNA was isolated by using an RNeasy mini kit (Qiagen). Reverse transcription was carried out by using the high-capacity cDNA kit (Applied Biosystems, Life Technologies). RT-PCR was performed on an Applied Biosystems 7500 real-time PCR system by using Taqman universal PCR master mix (Applied Biosystems) and FAM-TAM-labeled probes to measure expression of murine MULT-1 (*ulbp1*), Rae-1 family members (*rae1*), KC (*cxcl1*), LIX (*cxcl5*), and CCL2 (*ccl2*). Fold change in expression of mRNA was quantified by the 2^ΔΔCt^ method by using GAPDH as internal control (Applied Biosystems). PCR for the RSV L gene was performed, as described, normalized to 18s gene expression, and copy number was determined by using a plasmid that contains the RSV L gene as standard.^35^

### Protein measurements

Total serum IgE concentration was measured by using a total IgE ELISA kit (BD Biosciences). Antigen-specific serum IgE was measured as described.^36^ The BAL total protein concentration was assessed by Bradford assay (Sigma Aldrich).

### Statistical analysis

A group size of 5-6 mice was used. Unless otherwise indicated, data are expressed as mean (±SEM). Statistically significant differences between groups were determined by using a nonparametric Mann-Whitney *U* test. A *P* value of <.05 was accepted as significant. Results are indicated on figures as *P < .05; **P < .01, ***P < .001 or ns (not significant). PCR data were considered as normally distributed, and 1-way ANOVA with a Bonferroni *post hoc* test was used to assess significance. Graph generation and statistical analysis was performed by using GraphPad Prism software (version 5.00; GraphPad Software Inc, La Jolla, Calif). Data are representative of 2-4 independent experiments.

## Results

### NKG2D regulates HDM-allergic lung inflammation

**Fig E1:**
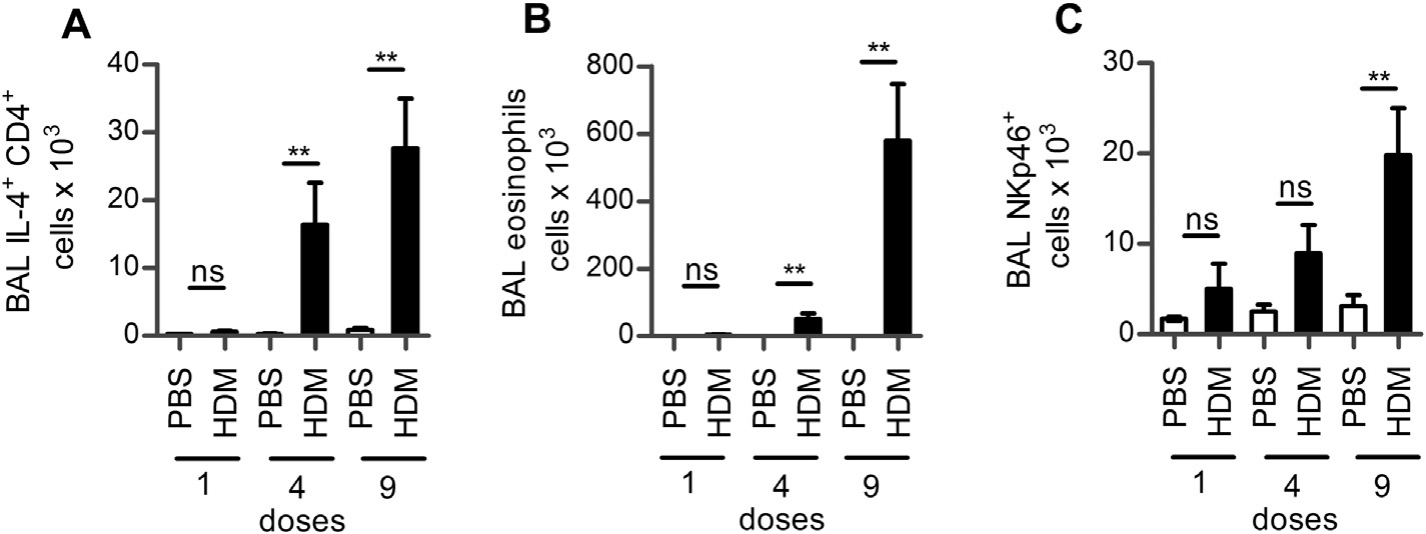
NKp46^+^ cell numbers in the airways over a time course of HDM-induced airway inflammation 9 doses of HDM were administered to BALB/c mice over 3 weeks to induce allergic airway inflammation. T_H_2 cell **(A)**, eosinophil **(B)**, andNKp46^+^**(C)** cell numbers in the airways after 1, 4, and 9 doses of allergen. *ns*, Not significant. **P < .01.

**Fig E2:**
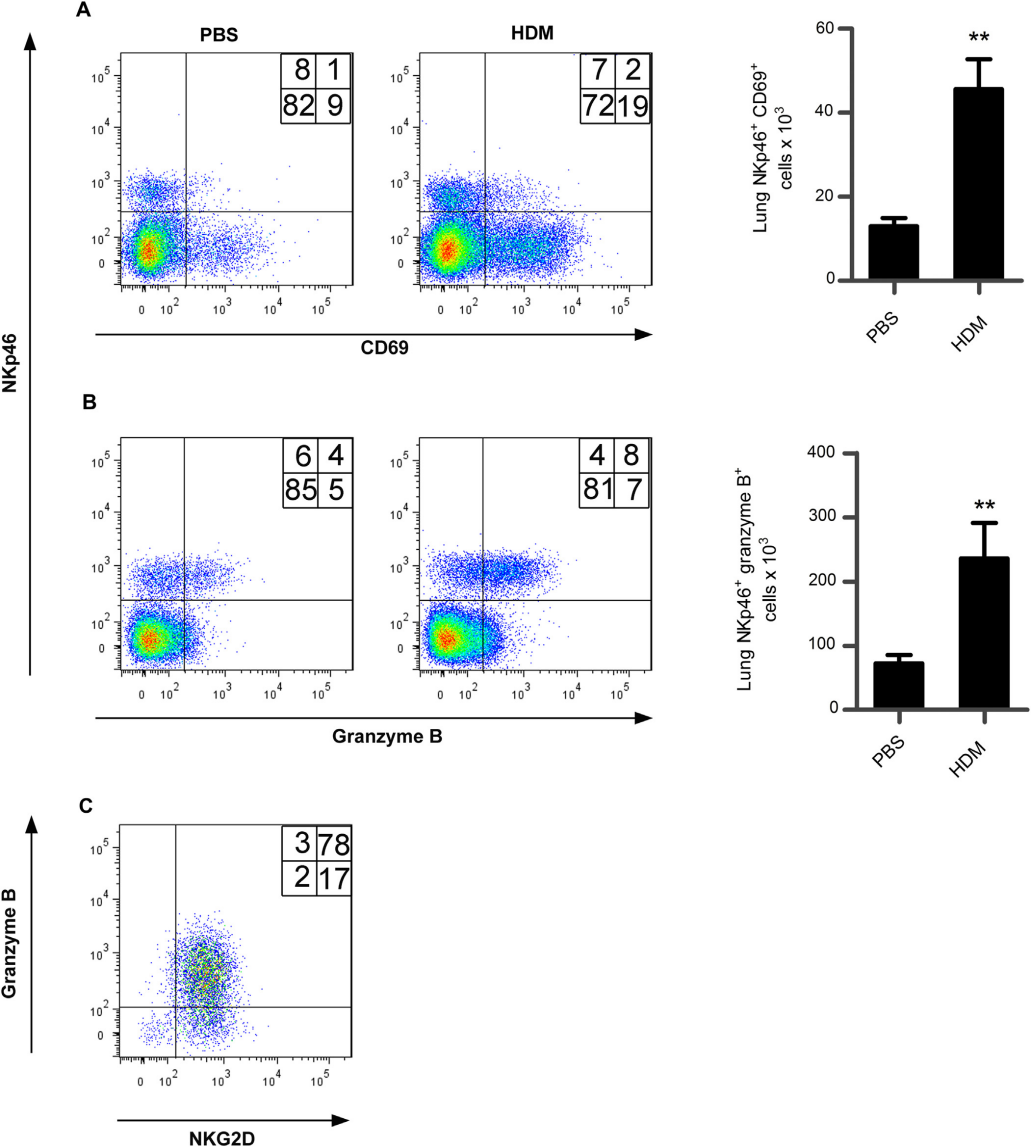
Phenotypic analysis of airway NKp46^+^ cells in HDM-allergic inflammation. **A,** CD69 expression in lung lymphocytes in PBS- and HDM-challenged C57/Bl6 mice, and the total number of lung NKp46^+^CD69^+^ lymphocytes. **B,** Granzyme B expression in lung lymphocytes in PBS- and HDM-challenged mice, and the total number of lung NKp46^+^granzyme B^+^ lymphocytes. **C,** Expression of NKG2D and granzyme B in NKp46^+^ cells in the lungs in HDM-allergic inflammation. **P < .01.

**Fig 1:**
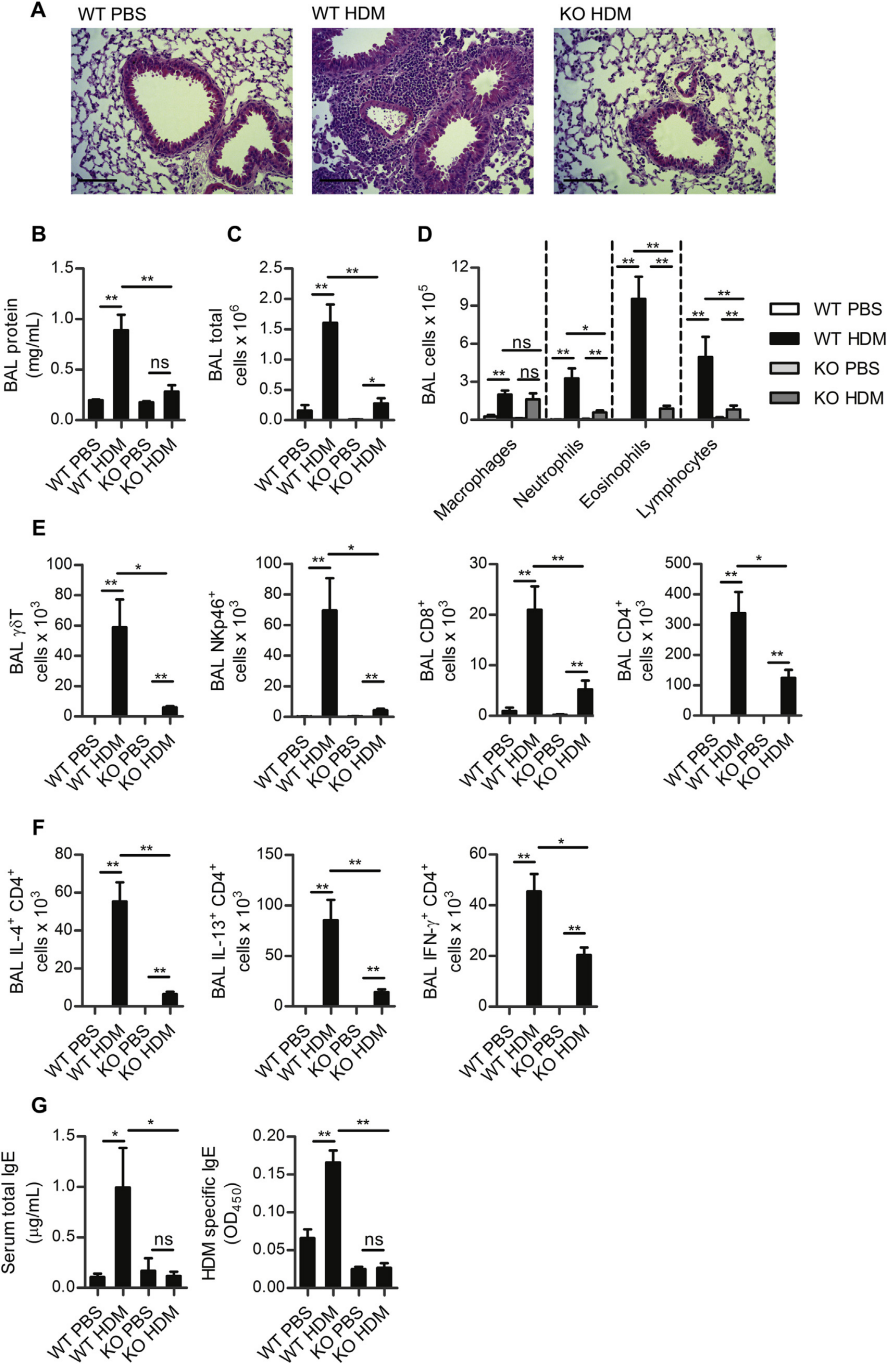
Inflammation in HDM-allergic airway disease is NKG2D dependent. **A,** Lungs of wild type *(WT)* or NKG2D-deficient *(KO)* mice challenged with PBS or HDM. **B,** BAL fluid protein concentration. **C,** Total number of airway cells. **D,** differential counts. **E,** Total airway γδTCR^+^, NKp46^+^, CD3^+^CD8^+^, and CD3^+^CD4^+^ lymphocytes. **F,** Total airway IL-4, IL-13, and IFN-γ^+^ CD4^+^ lymphocytes. **G,** Total concentration and HDM specific serum IgE. *Scale bar* = 100 μm. *ns*, Not significant. *P < .05 and **P < .01.

Allergic inflammation was induced in the airways of mice by intranasal inoculation of HDM extract 3 times a week over 3 weeks. This induces a pronounced eosinophilia and the recruitment of Th2 cells (CD4^+^IL-4^+^ and CD4^+^IL-13^+^ lymphocytes) to the airway.^33,34^ Although NK cells are present at low numbers in the airways and lung tissue in homeostasis, we found that they are greatly increased in number in allergic inflammation and that their increase in numbers in the airways over the course of induction of allergic inflammation paralleled development allergic inflammation (Fig E1 [in this article’s Online Repository at www.jacionline.org] and NKp46^+^CD3^−^ lymphocytes [Fig 1, *E*]). Further characterization of the NKp46^+^ airway lymphocytes in allergic inflammation revealed that these cells were activated (CD69 expression) and exhibited the NKG2D^+^ granzymeB^+^ phenotype of conventional, cytotoxic NK cells (Fig E2 in this article’s Online Repository at www.jacionline.org).

To determine the role of NKG2D in allergic lung inflammation, HDM allergen was administered to mice deficient in this receptor (*klrk1*^−/−^ mice). Remarkably, *klrk1*^−/−^ mice exhibited a profoundly impaired inflammatory response to allergen challenge compared with *klrk1*^+/+^ mice, with virtual abolition of the peribronchiolar inflammatory infiltrate seen in *klrk1*^+/+^ mice (Fig 1, *A*). Reflecting this reduced inflammation, the amount of total protein exudate into the airway, as measured in the BAL, did not increase in NKG2D-deficient mice (Fig 1, *B*) after allergen challenge. Furthermore, cellular recruitment was greatly attenuated in *klrk1*^−/−^ mice, specifically, there was a reduction in numbers of neutrophils, eosinophils, and lymphocytes in the airways compared with wild-type mice (Fig 1, *C* and *D*).

Within the lymphocyte populations, the numbers of γδT cells, NKp46^+^CD3^−^ lymphocytes, CD8^+^, and CD4^+^ T cells recruited to the airways were lower in *klrk1*^−/−^ mice (Fig 1, *E*). Furthermore, the total numbers of IL-4 and IL-13 secreting CD4^+^ cells in the airways were severely reduced (Fig 1, *F*) and within the recruited CD4^+^ T cells, the proportions secreting IL-4 and IL-13 were significantly lower in *klrk1*^−/−^ compared with *klrk1*^+/+^ mice (11.4% ± 2.4% vs 5.4% ± 0.7% and 22.2% ± 3.1% vs 11.7% ± 0.8%; respectively, mean ± SEM, *P* < .05). The total number of IFN-γ^+^ CD4^+^ cells were reduced in the airways of *klrk1*^−/−^ mice (Fig 1, *F*), but CD4^+^ lymphocyte IFN-γ production increased as a proportion of CD4^+^ cells (from 9% ± 1.6% to 19% ± 3%, mean ± SEM, *P* < .05). The proportion of IFN-γ secreting CD8^+^ cells in *klrk1*^−/−^ and *klrk1*^+/+^ mice was comparable (data not shown). Total serum IgE and HDM-specific IgE in *klrk1*^−/−^ mice was not elevated above that of naive mice (Fig 1, *G*). Importantly, changes in the inflammatory response seen in the airways were consistently reflected in changes to cell populations in the lung tissues (data not shown). Therefore, NKG2D deficiency leads to a profound impairment of HDM-allergic airway inflammation, and these data support a critical function for NKG2D in promoting this response.

### NKG2D does not alter inflammation and viral clearance in respiratory viral infection

**Fig E3:**
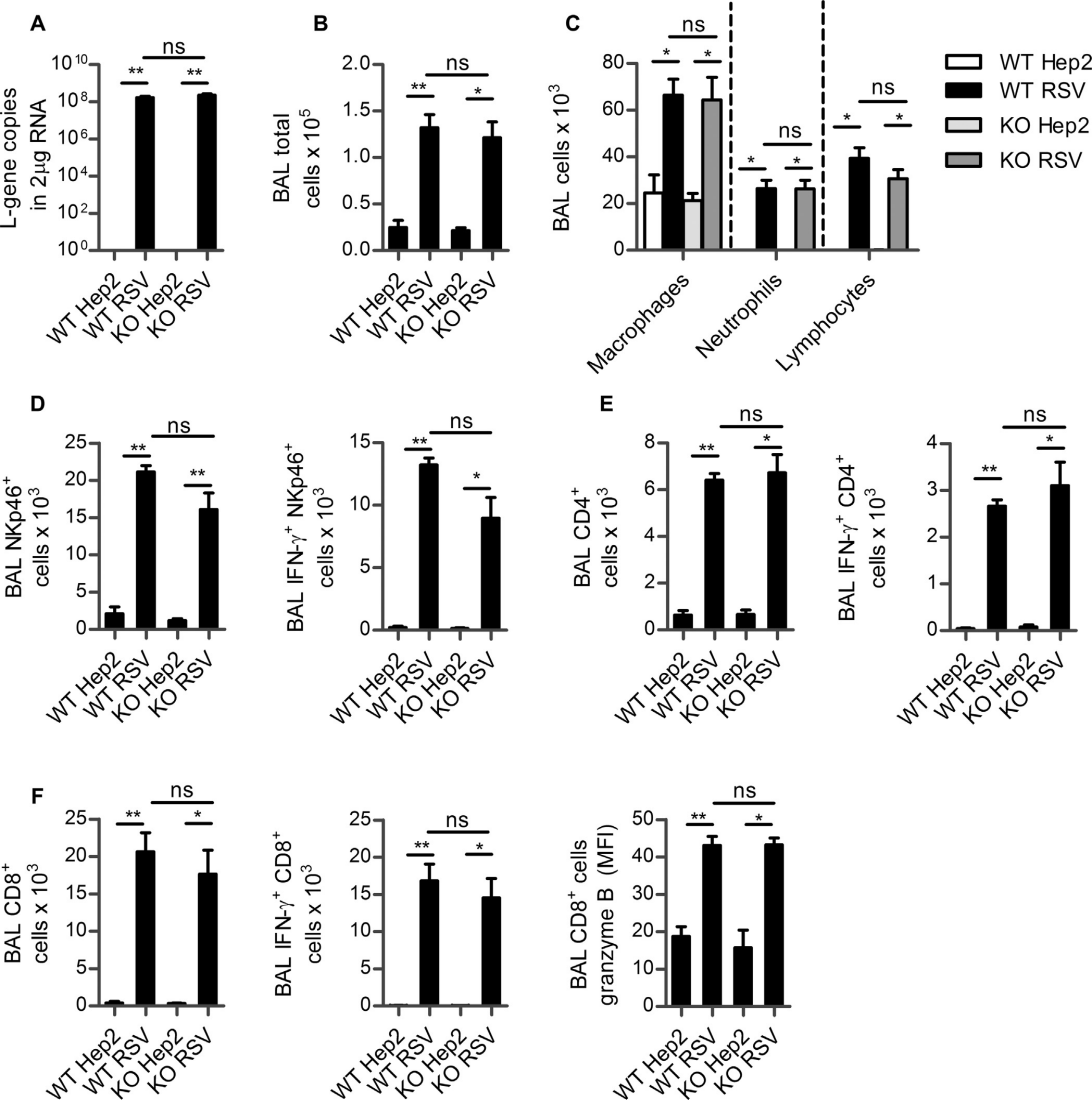
Immune responses to respiratory viral infection are NKG2D independent. Wild-type *(WT)* or NKG2D-deficient *(KO)* mice were infected with RSV. **A,** Lung viral load. **B,** Total airway cell number. **C,** Differential counts. **D,** Airway NKp46^+^ and NKp46^+^IFN-γ lymphocyte numbers. **E,** Airway CD4^+^ and CD4^+^ IFNγ^+^ lymphocyte numbers. **F,** CD8^+^ and CD8^+^IFNγ^+^ lymphocyte numbers and granzyme B expression in CD8^+^ lymphocytes. *ns*, Not significant. *P < .05 and **P < .01.

The *klrk1*^−/−^ mice used in this study exhibit only minor alteration in their NK cell receptor repertoire and respond normally to MCMV infection.^37^ Nevertheless, the attenuated inflammatory responses in NKG2D-deficient mice in response to HDM could reflect an alteration in NK-cell responsiveness or in immune responses in the lung. To address this, we infected mice with RSV and examined the viral load and immune response 4 days later. In this model, NK cells are recruited to the lungs within a few days of infection and promote the development of adaptive type 1 immunity.^38,39^ We detected no differences in viral load in *klrk1*^−/−^ mice, as measured by copies of viral L-gene at 4 days after infection, the peak of viral load in this model (see Fig E3, *A* in this article’s Online Repository at www.jacionline.org). Furthermore, there were no differences in responses to RSV infection in terms of total inflammatory cell recruitment into the airways (Fig E3, *B*) and the nature of the inflammatory infiltrate (Fig E3, *C*). NK cell (NKp46^+^CD3^−^ lymphocyte) numbers in the airways and their IFN-γ production was unaltered in *klrk1*^−/−^ mice compared with *klrk1*^+/+^ mice (Fig E3, *D*). Development of the CD4^+^ or CD8^+^ lymphocyte response was similarly unaltered (Fig E3, *E* and *F*). No IL-4 or IL-13 expressing lymphocytes were detected, and the RSV-induced weight loss did not differ between groups (data not shown). Analysis of these data demonstrates that, unlike HDM allergen challenge, after a respiratory viral infection, NKG2D-deficient mice are able to mount a robust inflammatory response in the lung, recruit NK cells, and control viral infection.

### NKG2D ligand MULT-1 is expressed in the lung in HDM-allergic inflammation

**Fig 2:**
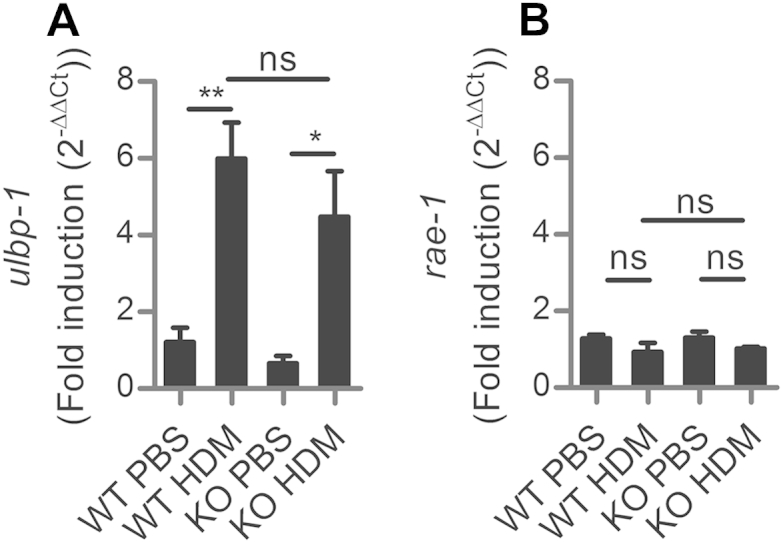
NKG2D ligand MULT-1 expression is upregulated in the lung during HDM-allergic inflammation. Mult-1 (*ulbp1*) **(A)** and *rae-1***(B)** gene family expression in whole lung in wild-type *(WT)* and NKG2D-deficient mice *(KO)* after 9 doses of HDM, as determined by RT-PCR. Expression is shown as fold change relative to PBS-challenged mice, normalized to GAPDH. *ns*, Not significant. *P < .05 and **P < .01.

To understand how NKG2D may be triggered after allergen challenge, we measured NKG2D ligand expression in lung tissue 24 hours after the final of 9 challenges with HDM extract. MULT-1 (*ulbp1*) and *rae1δ and rae1ε* are expressed in C57BL/6 mice, but H60 is not transcribed.^40^ Quantitative PCR demonstrated that there was localized, increased expression of the NKG2D ligand MULT-1 (*ulbp1*) but not Rae-1 family members, in the lung tissue (Fig 2, *A* and *B*). Increased expression of MULT-1 occurred equally well in *klrk1*^−/−^ mice as *klrk1*^+/+^. Therefore, the cell-stress response is activated in the lung after challenge with HDM allergen, but this is not NKG2D dependent.

### NKG2D does not regulate the early innate response to HDM

**Fig 3:**
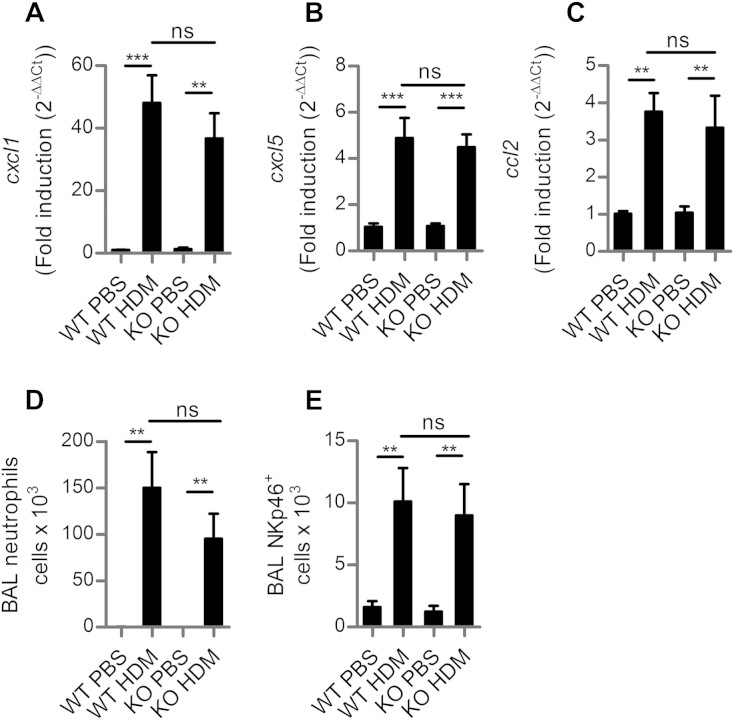
Inflammatory responses after a single dose of HDM are unaltered in NKG2D-deficient mice. Wild-type *(WT)* or *klrk1*^−/−^ mice *(KO)* mice received a single dose of HDM or PBS. *cxcl1***(A)**, *cxcl5***(B)**, and *ccl2***(C)** gene expression in the lung 24 hours after challenge relative to PBS-challenged mice. **D,** Numbers of airway neutrophils. **E,** NKp46^+^ cell numbers in the airways. *ns*, Not significant. **P < .01 and ***P < .001.

Early innate responses are triggered after detection of HDM allergen by resident lung cells, such as epithelial cells.^5^ Therefore, to determine the influence of NKG2D on early responses to allergen, we administered a single dose of HDM extract into the airways and assessed the inflammatory response 24 hours later. The *klrk1*^−/−^ mice expressed chemokines KC (*cxcl1)*, LIX (*cxcl5*), and MCP1 (*ccl2*) (Fig 3, *A*-*C*) and developed a marked neutrophilia of the airways to the same extent as *klrk1*^+/+^ mice (Fig 3, *D*). An early, localized recruitment of NK cells (NKp46^+^CD3^−^) occurred in the airways within 24 hours, which was unaltered by the absence of NKG2D (Fig 3, *E*). Thus, early expression of cytokine genes and inflammatory cell recruitment are not dependent on the presence of NKG2D.

### HDM allergic airway inflammation is regulated by NK-cell intrinsic NKG2D

**Fig 4:**
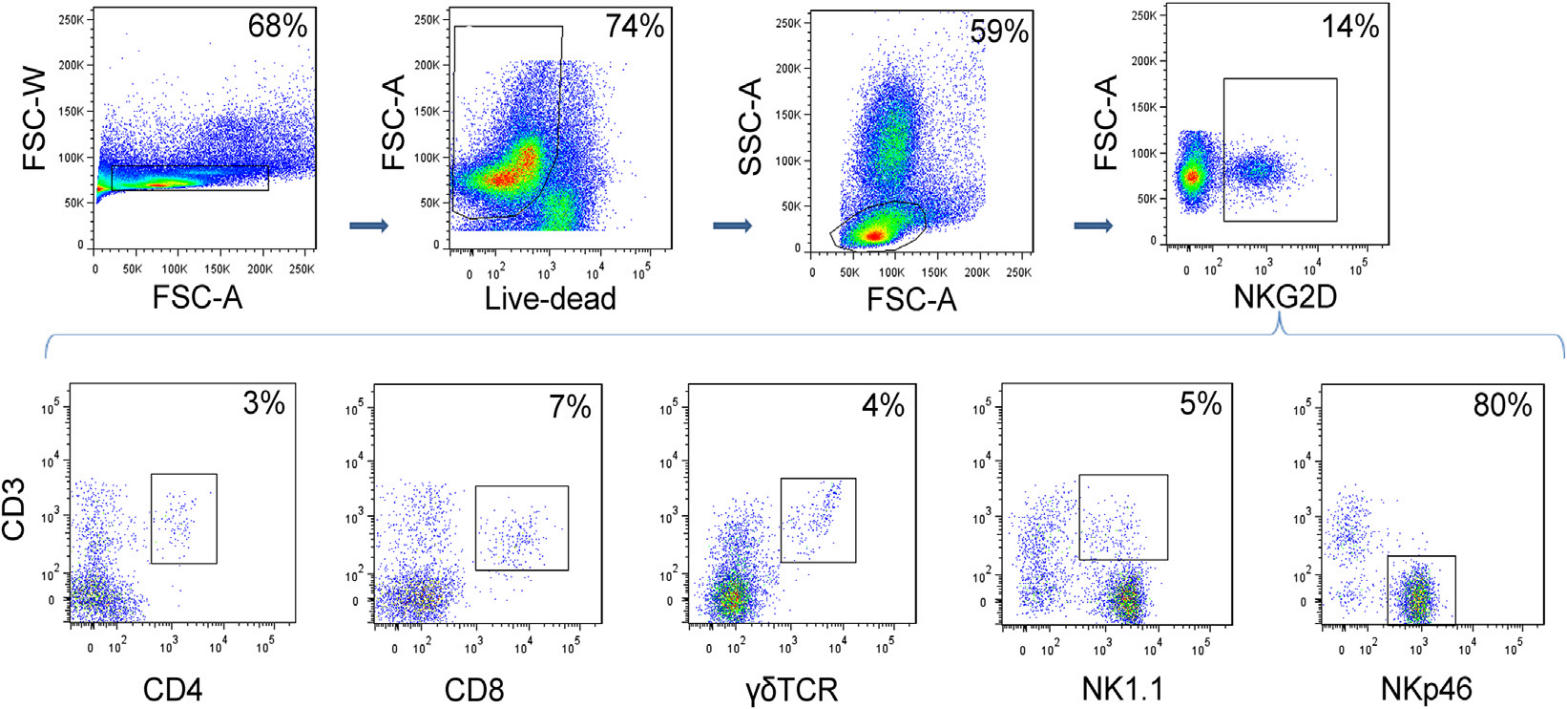
NK cells are the major NKG2D^+^ lymphocyte population in the lung in allergic inflammation wild-type C57/Bl6 mice received HDM to induce allergic airway inflammation. A single-cell suspension of lung tissue was analyzed by flow cytometry for NKG2D expression. Cells were gated on single cells and live lymphocytes, then for NKG2D^+^ cells. The proportion of NKG2D^+^ cells that are in each lymphocyte subset is shown in representative plots.

**Fig 5:**
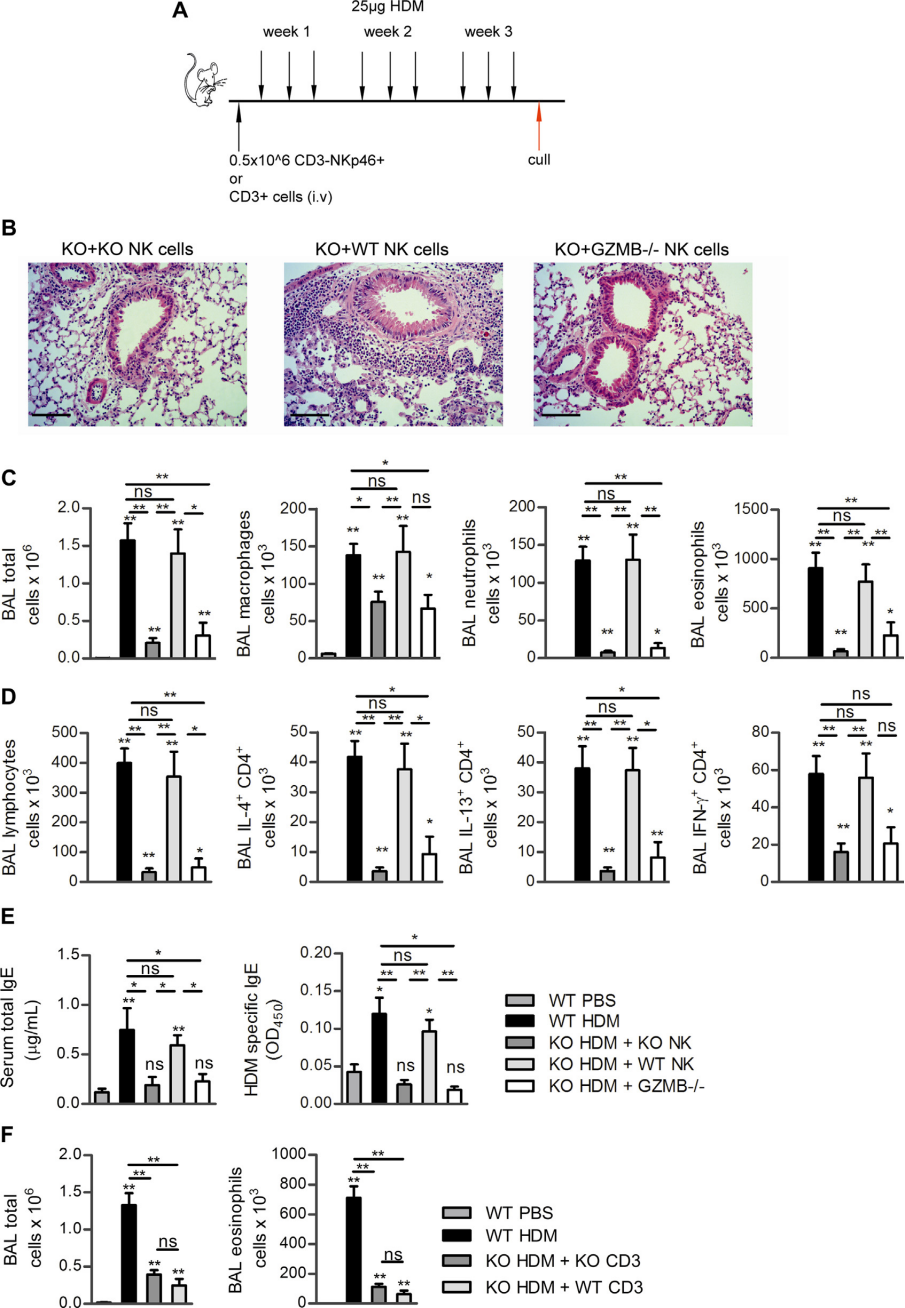
Restoration of airway inflammation by adoptive transfer of wild-type but not granzyme B deficient NK cells. **A,** Protocol for adoptive transfer. **B,** Lung histology of *klrk1*^−/−^ mice that received NK cells from *klrk1*^−/−^ mice (KO+KO); wild-type mice (KO+WT) or granzyme B deficient mice (KO+GZMB^−/−^). **C,** Total airway cells and differential counts. **D,** Airway lymphocyte numbers and cytokine-producing CD4^+^ cells. **E,** Serum IgE measurements. **F,** Adoptive transfer of CD3^+^ lymphocytes. *Scale bar* = 100 μm. *ns*, Not significant. *P < .05 and **P < .01.

To determine which cells express NKG2D in the airways in allergic inflammation, lung lymphocytes subsets were assessed by flow cytometry. Lung lymphocytes were first gated for NKG2D expression, and the constituent subpopulations were characterized. The majority (approximately 80%) of lymphocytes expressing NKG2D were NK cells (NKp46^+^CD3^−^) (Fig 4) but expression also was present on CD4^+^ and CD8^+^ T lymphocytes, and γδT cells and NKT cells. Thus, NK cells are the major lung population expressing the NKG2D activating receptor. To determine whether NK-cell or T-cell intrinsic NKG2D expression was sufficient to promote allergic inflammation, NKp46^+^CD3^−^ or CD3^+^ splenocytes were isolated from wild-type C57BL/6 mice and adoptively transferred into *klrk1*^−/−^ mice 24 hours before the first of 9 doses of HDM (Fig 5, *A*). The presence of transferred cells in the lung was confirmed 24 hours after the final HDM dose by enumerating NKG2D^+^ cells in lung tissue, and these constituted approximately 10% of NKp46^+^ and approximately 5% of CD3^+^ cells (data not shown).

Importantly, the transfer of wild-type NK cells was able to restore allergic inflammation to *klrk1*^−/−^ mice, which results in pronounced pulmonary inflammation (Fig 5, *B*) and inflammatory cell numbers in the airways were equal to that in HDM challenged *klrk1*^+/+^ mice (Fig 5, *C*). Wild-type NK cells were sufficient to restore airway macrophage, neutrophil, eosinophil, and lymphocyte recruitment to equal numbers and in equal proportions to that seen in *klrk1*^+/+^ mice (Fig 5, *C* and *D*). Furthermore, numbers of IL-4^+^, IL-13^+^, and IFN-γ^+^CD4^+^ cells also were equivalent to those present in the lungs of *klrk1*^+/+^ mice after transfer of NK cells into *klrk1*^−/−^ mice (Fig 5, *D*). Finally, serum total IgE and HDM-specific IgE were also restored to the level observed in *klrk1*^+/+^ mice when wild-type NK cells were adoptively transferred into *klrk1*^−/−^ mice (Fig 5, *E*). Importantly, transfer of *klrk1*^−/−^ NK cells did not restore allergic inflammation, which served as a control for any nonspecific effects of isolation and transfer, and nor did transfer of CD3^+^ splenocytes (which consisted of 56% CD4^+^, 37% CD8^+^, 3% γδTCR^+^, and 4% NKT cells) (Fig 5, *F*), which indicated that NK cell, but not T cell, intrinsic expression of NKG2D promotes HDM-allergic airway inflammation.

Granzyme B has been found to be elevated in the lungs of people with asthma after allergen challenge.^41,42^ To directly address the importance of granzyme B in NK-cell mediated allergic inflammation, NK cells from granzyme B deficient mice (*gzmb*^−/−^) were adoptively transferred into NKG2D-deficient mice to determine if the allergic phenotype would be restored. Interestingly, granzyme B deficient NK cells, similar to NKG2D-deficient NK cells, were unable to restore allergic inflammation (Fig 5, *B* to *E*), which indicated that both NKG2D expression and granzyme B production by NK cells are required to promote airway inflammation, T_H_2 cell recruitment to the lung and serum IgE.

## Discussion

In this study, we showed, for the first time, that NK cells promote allergic pulmonary inflammation in response to HDM allergen by mechanisms dependent on NK-cell intrinsic expression of the major activating receptor NKG2D and granzyme B. Mice that are deficient in NKG2D are resistant to HDM-allergic lung disease, which exhibit lower cell infiltration and reduced T_H_2 responses. NKG2D did not influence the innate response seen 24 hours after a single inoculation of HDM and was not required for immune responses to respiratory viral infection. Interestingly, adoptive transfer experiments demonstrated that both NKG2D and granzyme B expression by NK cells is required for the allergic response. Transfer of CD3^+^ lymphocytes did not restore allergic inflammation to NKG2D deficient mice, which suggests that NKG2D expression on T lymphocytes and NKT cells is not sufficient to promote allergic inflammation in this model. In summary, we describe specific, nonredundant functions for NK cell intrinsic NKG2D and granzyme B in HDM-allergic lung inflammation.

Previous work with murine models of allergic airway inflammation suggested a role for NK cells, but, because of the potential off-target effects of antibodies used for depletion, these studies are inconclusive.^43-49^ In our studies, we used NKp46 to isolate NK cells for adoptive transfer. In the spleen, this marker is exclusively expressed on NK cells,^50^ thus allowing conclusive demonstration that NK cells promote allergic lung inflammation in our model. Strid et al^30^ demonstrated a role for NKG2D expressed on intraepithelial lymphocytes in induction of allergy via the skin, and, when taken together with our data, this may suggest a general role for NKG2D in the promotion of allergy. We asked whether NKG2D was important for the adaptive or the innate response to HDM allergen. After a single challenge with HDM extract, we see no differences in NKG2D-deficient mice, which suggests that NKG2D is exerting its influence on the adaptive phase of the allergic response. Previous studies have shown that NK cells promote the adaptive T_H_1 response through the production of IFN-γ after migration to the lymph nodes^12^ and can produce type-2 cytokines,^51^ which contribute to inflammation in helminth infection.^14^ Our data demonstrate that NK-cell granzyme B rather than cytokine production, is required for HDM-allergic inflammation, although we do not exclude the possibility that NK cells making type-2 cytokines contribute to inflammation. The importance of granzyme B as an effector molecule suggests that cytotoxic mechanisms may play a role and NK cells could regulate allergic inflammation by targeting activated T cells, dendritic cells, or respiratory epithelial cells.^18,52-57^ Alternatively, granzyme B can regulate inflammation by noncytotoxic mechanisms, for example, IL-1α, which has undergone proteolysis by granzyme B, can promote allergic airway inflammation.^58-60^

Our demonstration of the importance of NK-cell activation in the HDM model of allergic airway disease is supported by studies in asthma, which suggests that our murine model is relevant to clinical disease. NK activity is high in the peripheral blood of people with asthma.^61-63^ Furthermore, in severe asthma, NK cells exhibit increased expression of CD69 and NKG2D, and expression levels of these surface molecules correlate with the percentage of peripheral blood eosinophils.^31^ After allergen challenge in patients with allergic asthma, granzyme B expressing NK cells and granzyme B protein are elevated in the airways.^41,42^ Furthermore, the NKG2D ligands MICA and ULBP-2, which can be cleaved from the cell surface and detected in soluble forms in the serum if produced in sufficient quantities, are elevated in the serum of children with HDM allergy.^32,64^ Based upon our findings, any factors that promote NK-cell activity would be predicted to worsen inflammation in asthma. NK cells are strongly activated by viral infection, and respiratory viral infections are the single leading cause of asthma exacerbations, which results in the major morbidity, mortality, and health care costs associated with asthma.^65^ Thus, NK-cell activation via NKG2D and their granzyme B production may be important new therapeutic targets in asthma and allergic disease.

**Key messages**

• Natural killer cell intrinsic expression of the activating receptor NKG2D is required for allergic pulmonary inflammation in response to house dust mite extract.
• Natural killer cells promote allergic pulmonary inflammation by production of granzyme B.
